# Impact of the malaria comprehensive case management programme in Odisha, India

**DOI:** 10.1371/journal.pone.0265352

**Published:** 2022-03-24

**Authors:** Madan M. Pradhan, Sreya Pradhan, Ambarish Dutta, Naman K. Shah, Neena Valecha, Pyare L. Joshi, Khageshwar Pradhan, Penny Grewal Daumerie, Jaya Banerji, Stephan Duparc, Kamini Mendis, Surya K. Sharma, Shiva Murugasampillay, Anupkumar R. Anvikar

**Affiliations:** 1 National Vector Borne Disease Control Programme, Government of Odisha, Bhubaneswar, India; 2 Indian Institute of Public Health, Bhubaneswar, India; 3 Kalinga Institute of Industrial Technology, Deemed to be University, Bhubaneswar, India; 4 University of North Carolina, Chapel Hill, North Carolina, United States of America; 5 National Institute of Malaria Research, New Delhi, India; 6 Independent Malariologist, Gallup, Washington, D.C., United States of America; 7 National Institute of Malaria Research Field Unit, Rourkela, India; 8 Medicines for Malaria Venture, Geneva, Switzerland; 9 Independent Malariologist, Colombo, Sri Lanka; 10 Global Public Health, Geneva, Switzerland; Freelance Consultant, Myanmar, MYANMAR

## Abstract

**Background:**

The Comprehensive Case Management Project (CCMP), was a collaborative implementation research initiative to strengthen malaria early detection and complete treatment in Odisha State, India.

**Methods:**

A two-arm quasi-experimental design was deployed across four districts in Odisha, representing a range of malaria endemicity: Bolangir (low), Dhenkanal (moderate), Angul (high), and Kandhamal (hyper). In each district, a control block received routine malaria control measures, whereas a CCMP block received a range of interventions to intensify surveillance, diagnosis, and case management. Impact was evaluated by difference-in-difference (DID) analysis and interrupted time-series (ITS) analysis of monthly blood examination rate (MBER) and monthly parasite index (MPI) over three phases: phase 1 pre-CCMP (2009–2012) phase 2 CCMP intervention (2013–2015), and phase 3 post-CCMP (2016–2017).

**Results:**

During CCMP implementation, adjusting for control blocks, DID and ITS analysis indicated a 25% increase in MBER and a 96% increase in MPI, followed by a –47% decline in MPI post-CCMP, though MBER was maintained. Level changes in MPI between phases 1 and 2 were most marked in Dhenkanal and Angul with increases of 976% and 287%, respectively, but declines in Bolangir (−57%) and Kandhamal (−22%). Between phase 2 and phase 3, despite the MBER remaining relatively constant, substantial decreases in MPI were observed in Dhenkanal (−78%), and Angul (−59%), with a more modest decline in Bolangir (−13%), and an increase in Kandhamal (14%).

**Conclusions:**

Overall, CCMP improved malaria early detection and treatment through the enhancement of the existing network of malaria services which positively impacted case incidence in three districts. In Kandhamal, which is hyperendemic, the impact was not evident. However, in Dhenkanal and Angul, areas of moderate-to-high malaria endemicity, CCMP interventions precipitated a dramatic increase in case detection and a subsequent decline in malaria incidence, particularly in previously difficult-to-reach communities.

## Introduction

Malaria remains a serious public health problem globally, with an estimated 241 million cases in 2020 [1]. India is one of eleven high malaria burden countries, carrying 1.7% of the global malaria burden, but 83% of the burden in the South East Asia region of World Health Organization [1]. Early detection and complete treatment (EDCT) remains a mainstay of malaria control and elimination programmes, along with vector control measures and surveillance [2,3]. Initially, EDCT is targeted at reducing morbidity and mortality, but ultimately aims to eliminate malaria by interrupting transmission through early identification of all cases and ensuring complete treatment of infected individuals. Programs and operational studies aimed at improving EDCT for malaria case reduction and elimination have been conducted in varies locations in India and other malaria endemic countries [2,4–11]. However, although there are commonalities in community-based organisation and the available tools, the political, cultural, geographical and economic challenges are often unique to the region and require the design and testing of specific interventions.

The eastern Indian state of Odisha has been one of the largest contributors to malaria case burden in India for past few decades. The state occupies only 4% of the Indian land mass and contains just 3% of its population but contributes around 40% of India’s annual malaria cases [12]. The humid climate, perennial streams, and forested hilly topography promotes malaria transmission and maintains malaria receptivity, leaving the region vulnerable to outbreaks and re-introduction where local elimination has been achieved. The topography also complicates access to malaria services, especially during the rainy season when many areas are inaccessible, which coincides with peak malaria transmission. For the first decade of the 21^st^ century, Odisha was grappling with a devastatingly high malaria burden. Intensified anti-malaria efforts in multiple fronts from 2008 to 2009 resulted in a significant decline in malaria cases by 2011–2013 [13]. However, from 2014 these successes diminished, with malaria incidence exceeding 2009 levels [14]. Thus, it became clear that EDCT had to be strengthened in Odisha.

The Comprehensive Case Management Project (CCMP), was a collaborative implementation research initiative of the Government of Odisha and the National Institute of Malaria Research (NIMR), financially and scientifically supported by Medicines for Malaria Venture (MMV). Launched in Odisha in early 2013, CCMP aimed to implement and then assess the impact of a repurposed and strengthened form of EDCT [15,16]. CCMP was piloted within programmatic settings, leveraging the existing state, district, and sub-district-level infrastructure of the National Vector-borne Disease Control Programme (NVBDCP), the umbrella programme that delivers malaria services in India. Overall, CCMP set out to strengthen universal and timely access to malaria diagnosis and treatment in different transmission settings against the backdrop of prevailing vector control measures [16]. To enable evaluation of the impact of CCMP, for every sub-district in which CCMP was implemented, one matched sub-district acted as a control and continued to receive routine NVBDCP services. In summary, CCMP was an active and responsive investigation into the operational feasibility of different approaches to implementing EDCT for malaria within the specific programmatic, environmental, and social context of Odisha.

A description of CCMP activities has been published [15,16]. An interim analysis indicated that CCMP resulted in an increase in malaria case reporting and improved detection which was likely to further lead to a subsequent decline in malaria case incidence [15,16]. This report presents an expanded and strengthened analysis comparing pre-CCMP, CCMP intervention, and post-CCMP monthly malaria detection and case incidence. Additionally, we report a detailed analysis of the impact of CCMP across four districts in Odisha with differing malaria endemicity: Bolangir has historically low malaria endemicity, with an annual parasite index (API) of 0–2 in 2010–2011, i.e. before the CCMP was implemented; Dhenkanal has moderate endemicity (API 2–10); Angul is a highly endemic region (API 10–20), and Kandhamal is hyperendemic (API >20). Notably, there were no reliable data on the *Plasmodium* species in these districts before CCMP was implemented, and presumptive treatment of fever with antimalarial drugs was still common, delivered mainly via the Accredited Social Health Activist (ASHA) network. Key challenges to implementing case management in these areas were poor geographical access, inadequate ASHA functioning, poor surveillance capabilities, limited supervision, insufficient stocks of commodities at the ASHA level, programmatic issues from competing healthcare programs, limited vector control, poor follow-up to confirm treatment completion, and disjointed data management systems.

Understanding the influence of the CCMP interventions across areas of differing malaria endemicity has allowed greater refinement of the malaria control and elimination program in Odisha, directed at reducing transmission and ultimately malaria elimination.

## Materials and methods

### Study design

**Fig 1:**
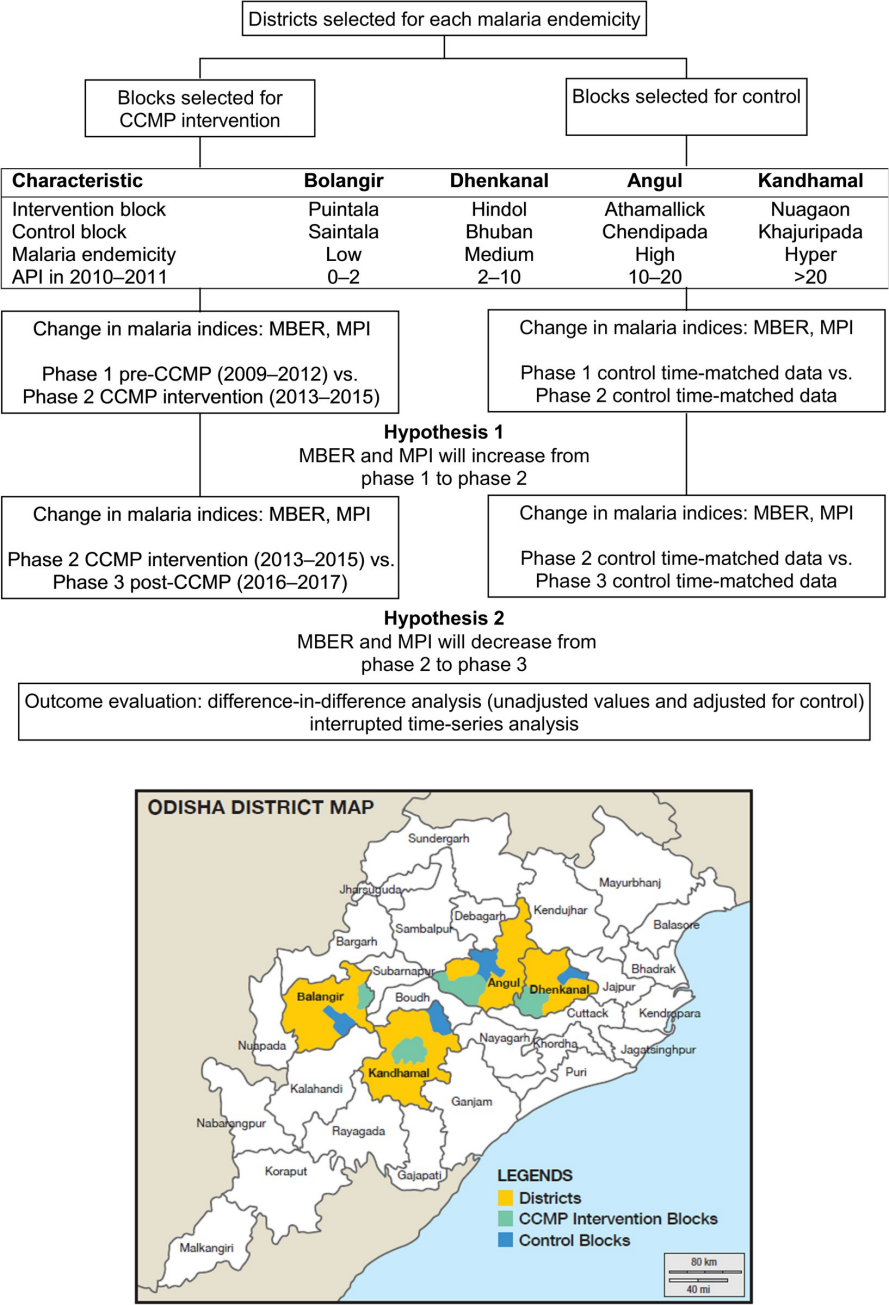
Study design and location of CCMP and intervention blocks. API, annual parasite index = total number of positive slides for parasite in a year x1000 / total population; CCMP, Comprehensive Case Management Project; MBER, monthly blood examination rate = total number of slides examined in a month x1000 / total population; MPI, monthly parasite index = total number of positive slides for parasite in a month x1000 / total population.

CCMP was a two-arm quasi-experimental implementation research study deployed across four districts in Odisha, representing a range of malaria endemicity: Bolangir (low), Dhenkanal (moderate), Angul (high), and Kandhamal (hyper) (Fig 1). From each district one intervention block one control block were selected, with control blocks matched based on malaria incidence in 2010–2011. A block is the second tier of general administration and primary health care in India, lying above the village-level and catering to roughly 100,000 people. For villages in both the control and intervention blocks, key malaria transmission risk factors were collected. This comprised the location of the village, the distance from an ASHA or any other public health facility or provider, access to the village by road, proximity to irrigation canals and natural streams, cultivation, and forest coverage. The control blocks received routine NVBDCP-prescribed malaria control measures, but none of the additional inputs received by the CCMP intervention blocks.

### CCMP interventions

Following the identification of barriers to case management, CCMP implementation focused on improving the quality of malaria services, promoting universal coverage of malaria diagnosis and treatment, and enhanced disease surveillance. The programme has been described previously in detail [16]. Briefly, the key components of the package included 1) training and supportive supervision of ASHAs; 2) identification and mapping of hamlets not covered by resident ASHAs and recruitment and training of alternative providers residing in these areas; 3) introduction of patient cards for tracking and follow up of all malaria positive patients until treatment completion, and monitoring for adverse reactions; 4) creation of additional microscopy centres at the sub-block levels, which also served as monitoring and supportive supervisors for village level providers; 5) mass screening and treatment (MSAT) in the high endemic hard-to-reach ‘pockets’ prior to malaria transmission seasons; 6) ensuring an uninterrupted supply of rapid diagnostic tests (RDTs) and antimalarial drugs at the village level through strengthening the supply chain; 7) enhancing malaria surveillance by the appointment of personnel at the sub-district level with responsibility for surveillance and maintenance of patient line lists at village level derived from the standard NVBDCP forms; and 8) introduction of an electronic health information management system (DHIS2: www.dhis2.org) to direct evidence-based programmatic actions [16].

### Analysis population

All of the resident population in the control and intervention blocks was included in the study. Malaria patients included in the analysis were of any age or sex presenting at any level of institution with fever and with malaria confirmed by RDT or microscopy. Microscopists underwent 28 days of training at the Regional Office of Health and Family Welfare as well as on-the-job training and support. Newly qualified microscopists were required to validate their findings with an RDT. All malaria positive slides and 10% of negative slides were sent to the district headquarters for quality control. Individuals with a positive malaria test were treated according to the National Malaria Guidelines current at the time of their treatment [17], and followed up to encourage treatment completion.

### Study period

To assess the final impact of CCMP in the intervention blocks across the four districts, two hypothesized inflection points were chosen: 2013 marking the beginning of the surge in malaria case reporting with the roll out of CCMP, with 2016 marking the beginning of the decline in malaria incidence. Consequently, three phases were identified: phase 1 pre-CCMP (2009–2012), phase 2 CCMP intervention (2013–2015) and phase 3 post-CCMP (2016–2017).

### Data collection

Data on the number of blood slides examined, RDTs performed, malaria patient details, treatment and follow-up were captured on malaria case forms. Malaria case forms of the routine programme were checked for completeness and accuracy by the block level manager (BLM). Data were captured using spreadsheets and imported into a malaria module based on the open-source health information system DHIS2 (www.dhis2.org). The data were stored in the database administered by the state NVBDCP for the entire period of CCMP (2013–2017), which was secured by using digital passwords known to authorised staff and used for tracking patients, when needed. Capacity building to train CCMP staff on data entry and validation was conducted, as well as training on data analysis and generating the required indicators and what action should be taken based on such indicators. In the intervention blocks, patient cards were issued to all confirmed malaria cases, with unique identification numbers. The patient cards were devised to track treatment compliance, identify any adverse events early, and identify relapses or recurrences.

Block population data was initially collected from the census (2011) system of India and then updated using the population denominator as reported by the NVBDCP over the year. Routine NVBDCP data comprised block-wise monthly blood examination rate (MBER) and monthly parasite index (MPI) extracted from the NVBDCP information system for both the intervention and control blocks from 2009 through 2017. MBER was defined as the number of blood samples examined for malaria by RDT or microscopy every month amongst the population under surveillance and was considered a surrogate for programmatic coverage. MPI was defined as the monthly diagnosis of parasitologically confirmed malaria cases in the population under surveillance and was considered as a marker for malaria incidence.

### Statistical analysis

Two sets of analyses were carried out–the first comparing phases 1 and 2 (pre-CCMP and CCMP intervention). This replicates the interim analysis [16], except conducted at the block-level, as well as overall. The second analysis compared phases 2 and 3 (CCMP intervention and post-CCMP). It is key that MBER and MPI are considered together, as the detection of malaria cases (MPI) is dependent upon the programmatic coverage (MBER). Two hypothesis were considered: 1) that phase 2 experienced a surge in MBER and MPI compared to phase 1; and 2), that phase 3 experienced a decline in MBER and MPI compared to phase 2 (Fig 1). Data were analysed using difference-in-difference analysis and interrupted time-series analysis.

Difference-in-difference analysis. A difference-in-difference (DID) framework was used to analyse the data. The outcome was considered as a count variable (either MBER or MPI) and the basic equation of the Poisson regression to model the count data was used:

$$Log(Y)=\beta_{0}+\beta_{1}x_{1}+\beta_{2}x_{2}+\beta_{3}x_{1}*x_{2}+offset(log(population))+\varepsilon$$

Where: *Y* = count of outcome indicator; *x*_1_ = time period / intervention phase expressed as 0, 1; with 0 indicating phase 1 in a phase 1 versus phase 2 comparison, and 1 indicating phase 2 in a phase 2 versus phase 3 comparison; *x*_2_ = intervention status expressed as 0, 1; with 0 indicating the control block, and 1 indicating the intervention block. *x*_1_ * *x*_2_ = the interaction between phase and the intervention status; Log(population) of the block was used as the offset variable in the equation. *β*_0_ *β*_1_ *β*_2_ *β*_3_ were all unknown parameters to be estimated and *ε* was a random, unobserved ‘error’ term. The coefficients of interest were: *β*_0_ = intercept; *β*_1_ = difference between pre- and post-intervention in the control block; *β*_2_ = difference between intervention and control in the pre-intervention phase and; *β*_3_ = impact of intervention (difference-in-difference). All the coefficients were anti-logged to derive the risk ratio.

Interrupted time-series analysis. Block-wise panel data (time-series data), of epidemiological indicators (MBER and MPI), were analysed using a segmented regression framework, i.e. ‘interrupted time-series analysis (ITS)’. The ITS analysis validates the underlying assumption of parallel trends of DID analysis and complements its findings. Through the ITS framework, count outcome data were regressed over time to estimate the ‘trends’ in MBER and MPI (long term effects); and trends interrupted by inflection points to estimate the changes in ‘levels’ (immediate effect). Using ITS analysis, the changes in both trends and levels comparing phase 1 to phase 2 and phase 2 to phase 3 were examined. For the comparison of phase 1 and 2, a change in level was defined as the difference between the observed level at the first intervention time point and that predicted by the pre-intervention time trend, and a change in trend was defined as the difference between post- and pre-intervention slopes [18]. Differences in trends and levels between phases 2 and 3 were defined similarly. These estimates were adjusted for corresponding changes in the control block. The ITS regression equation was:

$$Log(Y)=\beta_{0}+\beta_{1}x_{1}+\beta_{2}x_{2}+\beta_{3}x_{3}+\beta_{4}x_{1}*x_{2}+\beta_{5}x_{2}*x_{3}+\beta_{6}x_{1}*x_{3}+\beta_{7}x_{1}*x_{2}*x_{3}+offset(\log\left(population\right))+\varepsilon$$

Where: *Y* = count of outcome indicator; *x*_1_ = time as a continuous variable since the start of the data collection; *x*_2_ = phase expressed as 0, 1; with 0 indicating phase 1 in a phase 1 versus phase 2 comparison and 1 indicating phase 2 in a phase 2 versus phase 3 comparison; *x*_3_ = intervention status expressed as 0, 1, with 0 indicating the control block, and 1 indicating the intervention block;*x*_1_ * *x*_2_; *x*_2_ * *x*_3_; *x*_1_ * *x*_3_ = two-way interactions between the control block and intervention block; *x*_1_ * *x*_2_ * *x*_3_ = three-way interaction between time, phase and intervention status; Log(population) of the block was used as the offset variable in the equation. The coefficients of interest in ITS were: *β*_6_ = difference in *levels* between phases in intervention (control-adjusted) and; *β*_7_ = difference in *trends* between phases in intervention (control-adjusted). All the coefficients were anti-logged to derive the risk ratio.

### Ethics statement

The study was approved by the Institutional Ethics Committee of ICMR-National Institute of Malaria Research. As anonymised aggregated data was analysed, informed consent was not required.

## Results

### Malaria risk factors

The CCMP intervention blocks had worse scores than the control blocks on most malaria risk factors, including road access (61% vs. 72%), denser forest cover (37% vs. 16%), situated in foothills (34% vs. 21%) and further away from the designated diagnostic facility (1.7 km vs. 1 km). Overall, three of the four control areas had lower malaria risk factors than the intervention areas. However, the control and intervention areas were still comparable in regard to their relative differences and the transmission risk factors varied substantially between the districts (S1 Table).

### Study population and patients

Based on the Indian 2011 census, 99.4% (888,528/894,159) of the study population was classified as living in rural areas, with 49.3% (441,029/894,159) females and 50.7% (452,930/894,159) males, and 12.5% (111,489/894,159) aged 0–6 years (S2 Table). In 2013, the first year of the CCMP intervention, the study population included 483,849 individuals in the intervention blocks and 448,594 across the control blocks (S3 Table). Between January 2013 and December 2013, there were 73,873 malaria tests conducted in the intervention blocks of which 4,793 (6.5%) were positive, and in 2016, 113,727 tests were done of which 4,046 (3.6%) were positive (S3 Table). For the intervention blocks, the proportion of patients that had complete patient cards increased from 44.5% (2135/4793) in 2013 to 100% (4046/4046) in 2016 and the proportion of patients with complete follow up increased from 43.1% (2065/4793) in 2013 to 98.9% (4002/4046) in 2016, with this trend observed across all the intervention blocks (S4 Table).

### Impact analysis

#### Difference-in-Difference (DID) analysis

**Fig 2:**
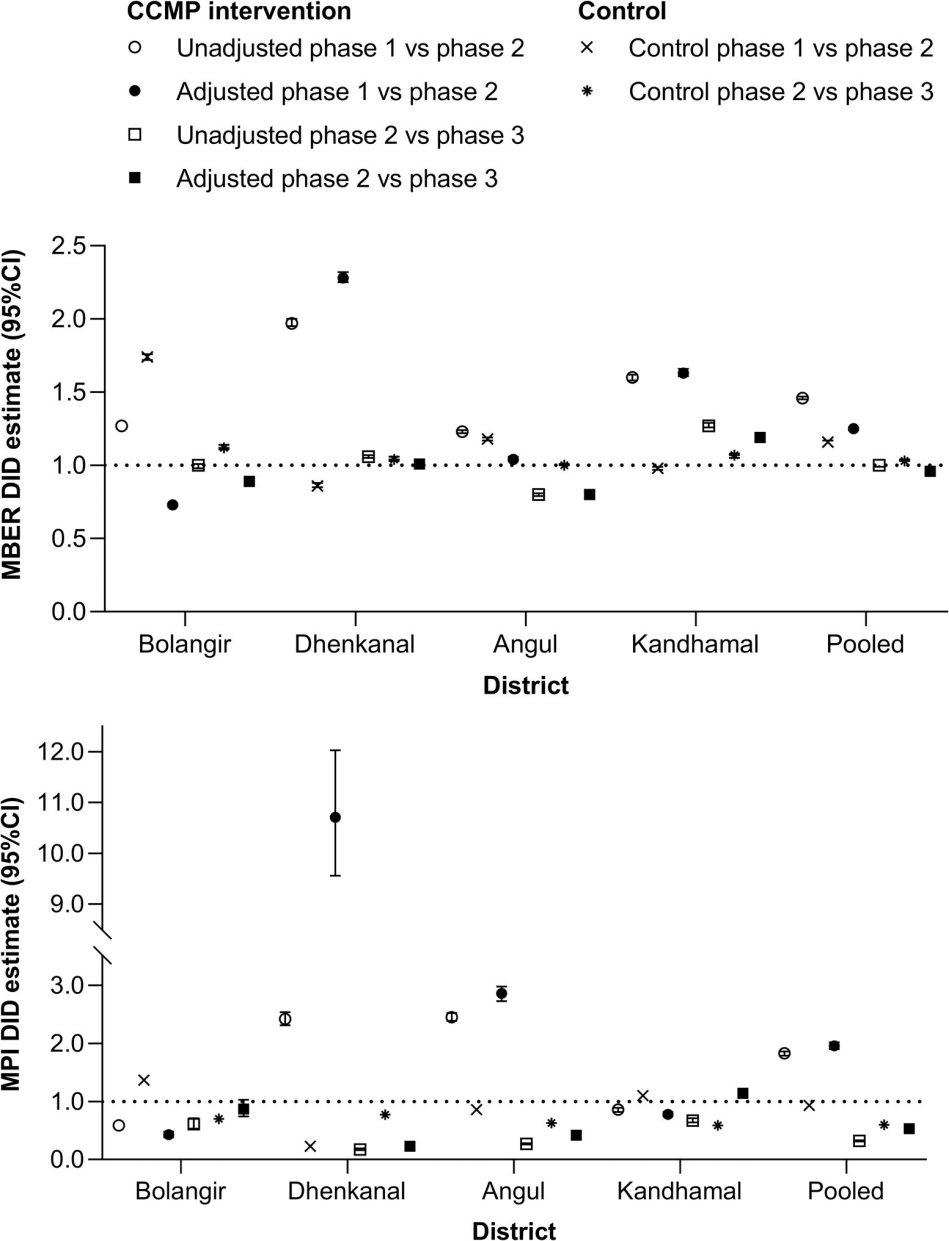
Difference-in-difference (DID) estimates for MBER and MPI in CCMP intervention blocks. Data are shown as CCMP intervention block unadjusted values and adjusted for contemporaneous changes in the control blocks; a value of 1 indicates no change, less than 1 is a decrease and more than 1 represents an increase. CCMP, Comprehensive Case Management Project; MBER, monthly blood examination rate = total number of slides examined in a month x1000 / total population; MPI, monthly parasite index = total number of positive slides for parasite in a month x1000 / total population.

Results of the DID analysis are shown in Fig 2 and S5 Table. Following adjustment for changes in the control blocks, in the CCMP intervention blocks the pooled MBER was 25% higher (95%CI 24, 26) in phase 2 compared to phase 1. There was also a 93% increase (95%CI 91, 95) in adjusted pooled MPI. Thus, the investigation of cases and malaria detection rate increased overall from phase 1 to phase 2, supporting hypothesis 1 (Fig 1). In phase 3 compared to phase 2, the adjusted pooled MBER was almost unchanged with a small decline (−4%; 95%CI −4, −3). However, the adjusted pooled MPI declined by −47% (95%CI −49, −45). These results support hypothesis 2 weakly for MBER, which remained steady, and strongly for MPI (Fig 1). Thus, although the investigation of malaria cases remained similar post-intervention versus during CCMP, the malaria case identification rate declined, suggesting a reduction in malaria prevalence.

There was considerable variation between the individual districts (Fig 2, S5 Table), with adjusted MBER increasing by a maximum of 128% (95%CI 125, 132) in Dhenkanal (moderate endemic), with smaller increases in other districts, except for Bolangir (low endemic), where a −27% (95%CI −28, −26) decline in MBER was observed. Adjusted MPI increased more than ten-fold in Dhenkanal, and by nearly 3-fold in Angul (high endemic), but declined in both Kandhamal (hyperendemic) and Bolangir. Between phase 2 and phase 3, the maximum adjusted increase in MBER was in Kandhamal (19%; 95%CI 17, 21) and the maximum adjusted decrease was in Angul (−20%; 95%CI −22, −19). In contrast, the MPI declined from phase 2 to 3 in all districts, with a maximum adjusted decline in Kandhamal (−41%; 95%CI −42, −38) (Fig 2, S5 Table).

#### Interrupted time-series (ITS) analysis

**Fig 3:**
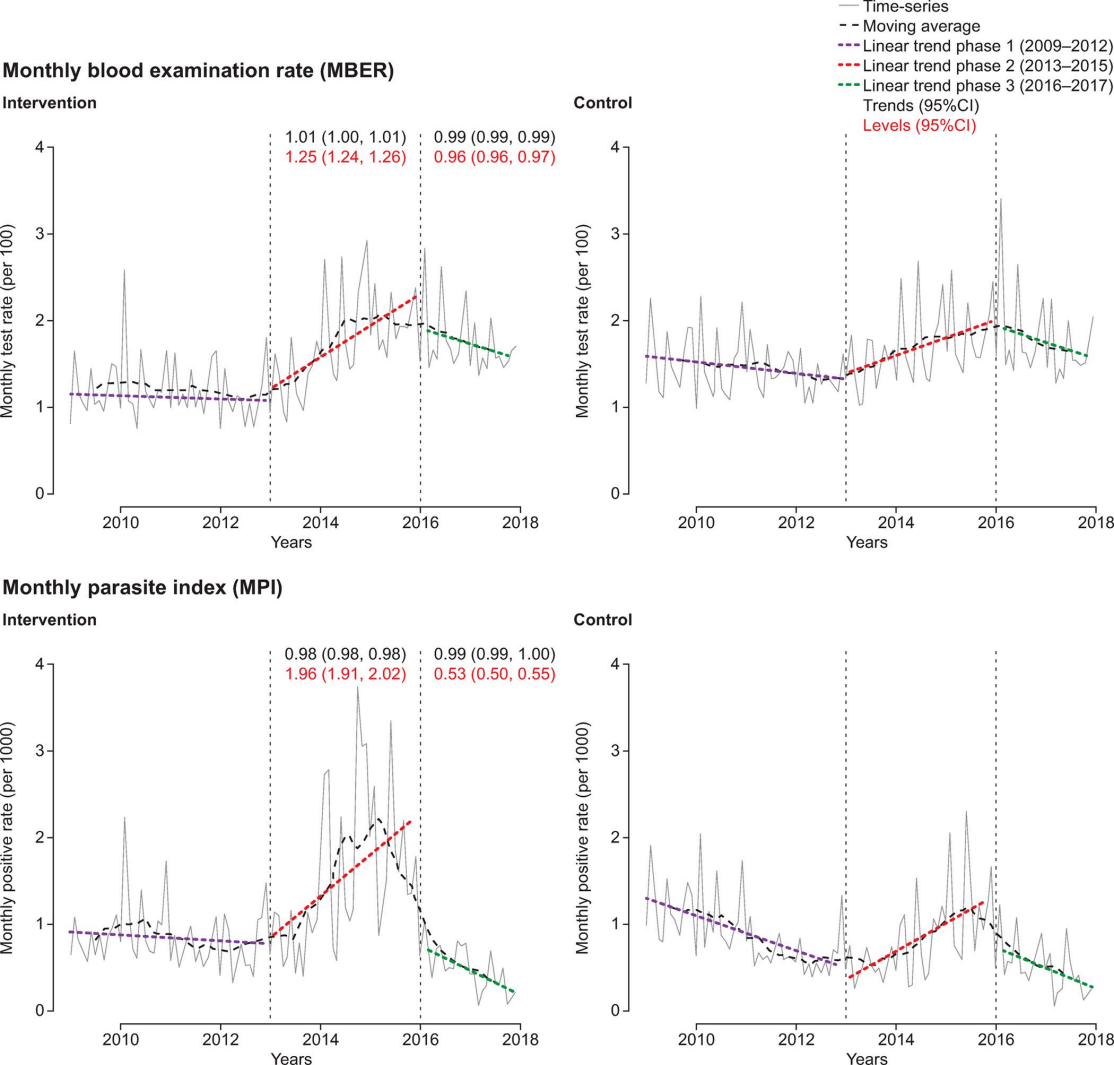
Interrupted time-series analysis of changes in malaria indices for pooled data. MBER, monthly blood examination rate = total number of slides examined in a month x1000 / total population; MPI, monthly parasite index = total number of positive slides for parasite in a month x1000 / total population.

For the pooled ITS analysis, the adjusted *trends* in MBER and in MPI remained essentially unchanged between phases 1 and 2 and between phases 2 and 3 (Fig 3, S6 and S7 Tables). However, there was a significant control-adjusted increase in MBER *level* (25%; 95%CI 24, 26) and in MPI level (96%; 95%CI 91, 102) between phases 1 and 2. Between phases 2 and 3, there was a slight decrease in adjusted pooled MBER level (−4%; 95%CI −4, −3) but a substantial decrease of −47% (95%CI −50, −45,) in adjusted pooled MPI level (Fig 3, S6 and S7 Tables).

**Fig 4:**
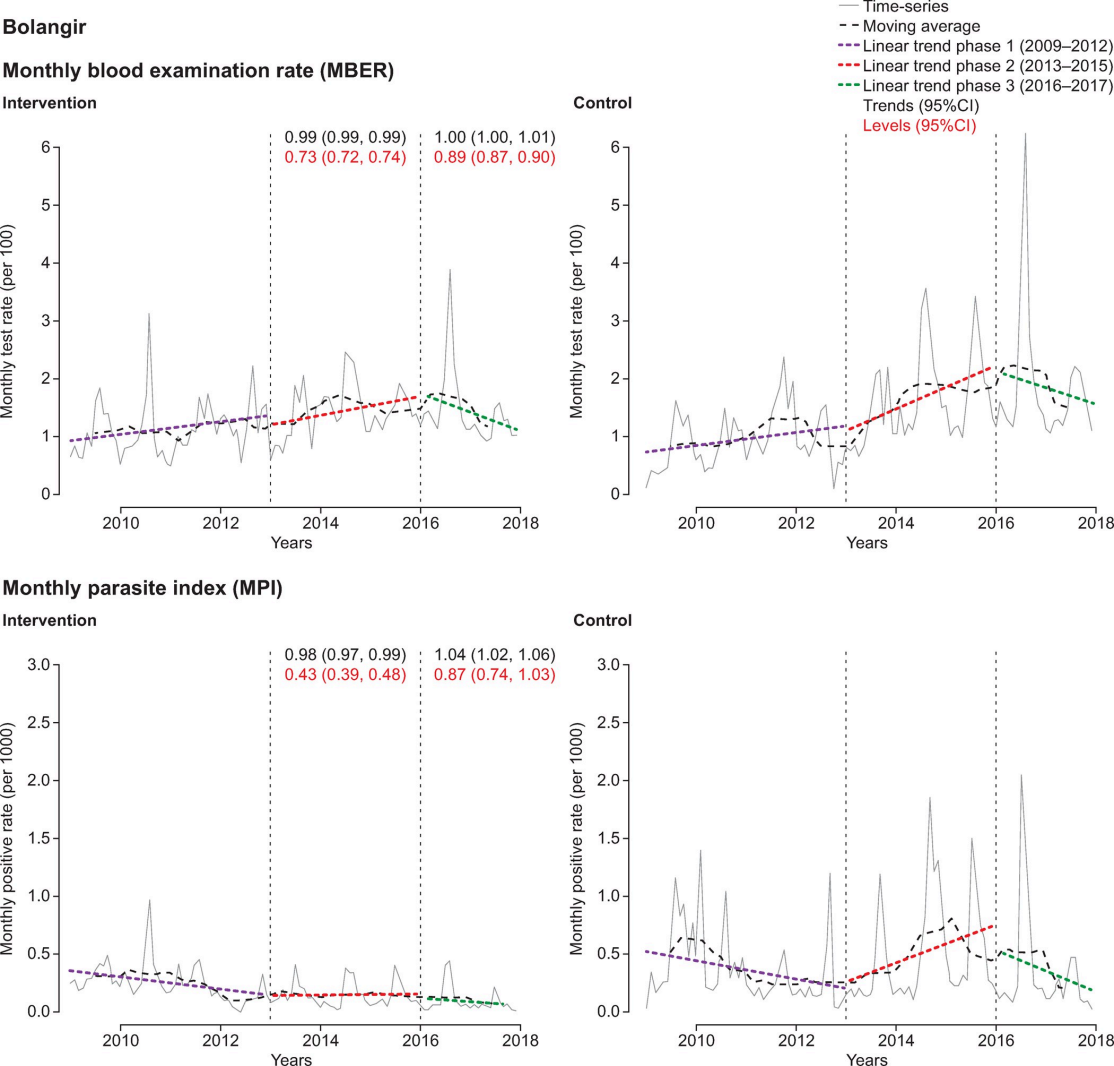
Interrupted time-series analysis of changes in malaria indices for Bolangir. MBER, monthly blood examination rate = total number of slides examined in a month x1000 / total population; MPI, monthly parasite index = total number of positive slides for parasite in a month x1000 / total population.

**Fig 5:**
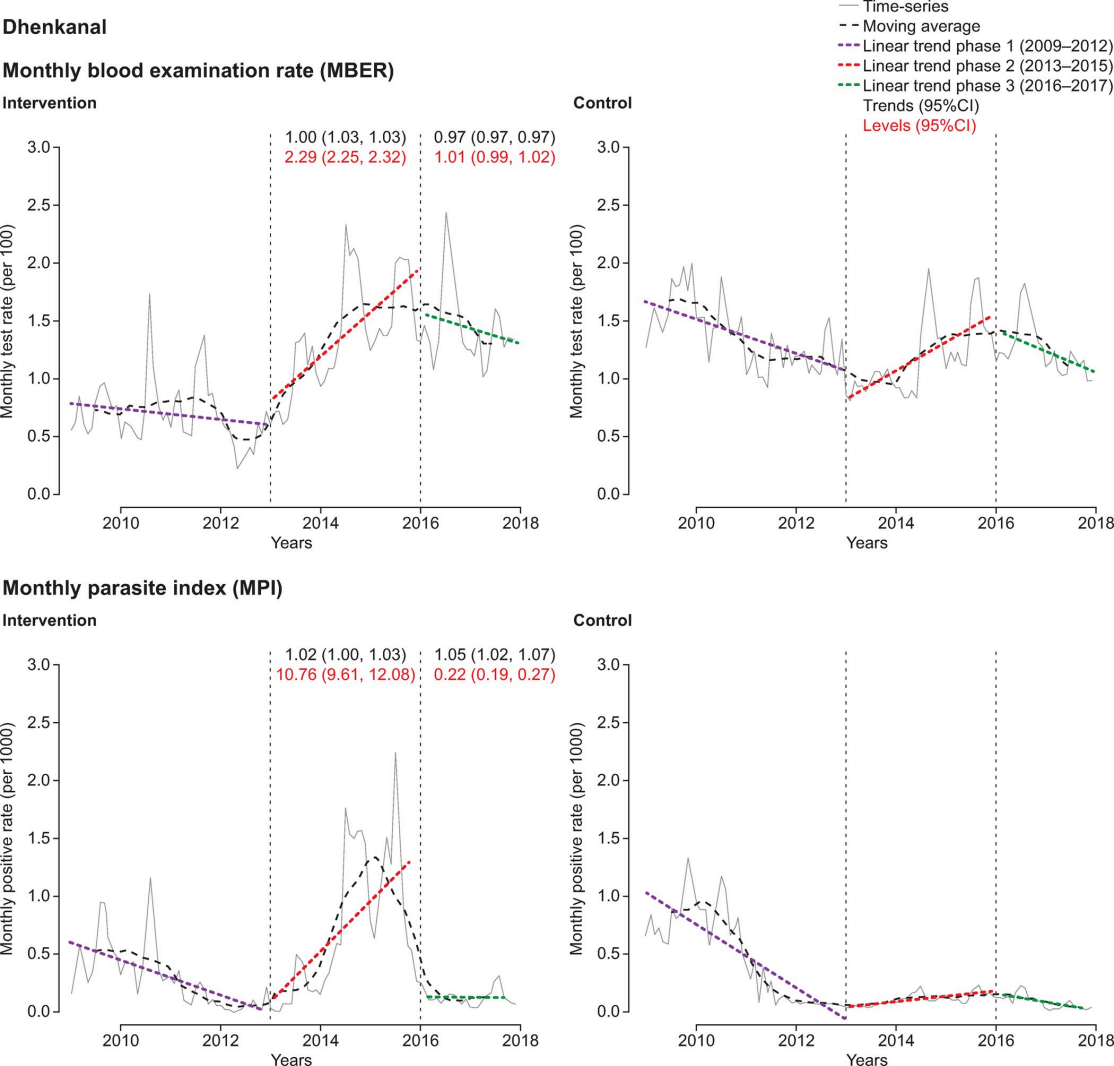
Interrupted time-series analysis of changes in malaria indices for Dhenkanal. MBER, monthly blood examination rate = total number of slides examined in a month x1000/total population; MPI, monthly parasite index = total number of positive slides for parasite in a month x1000 / total population.

**Fig 6:**
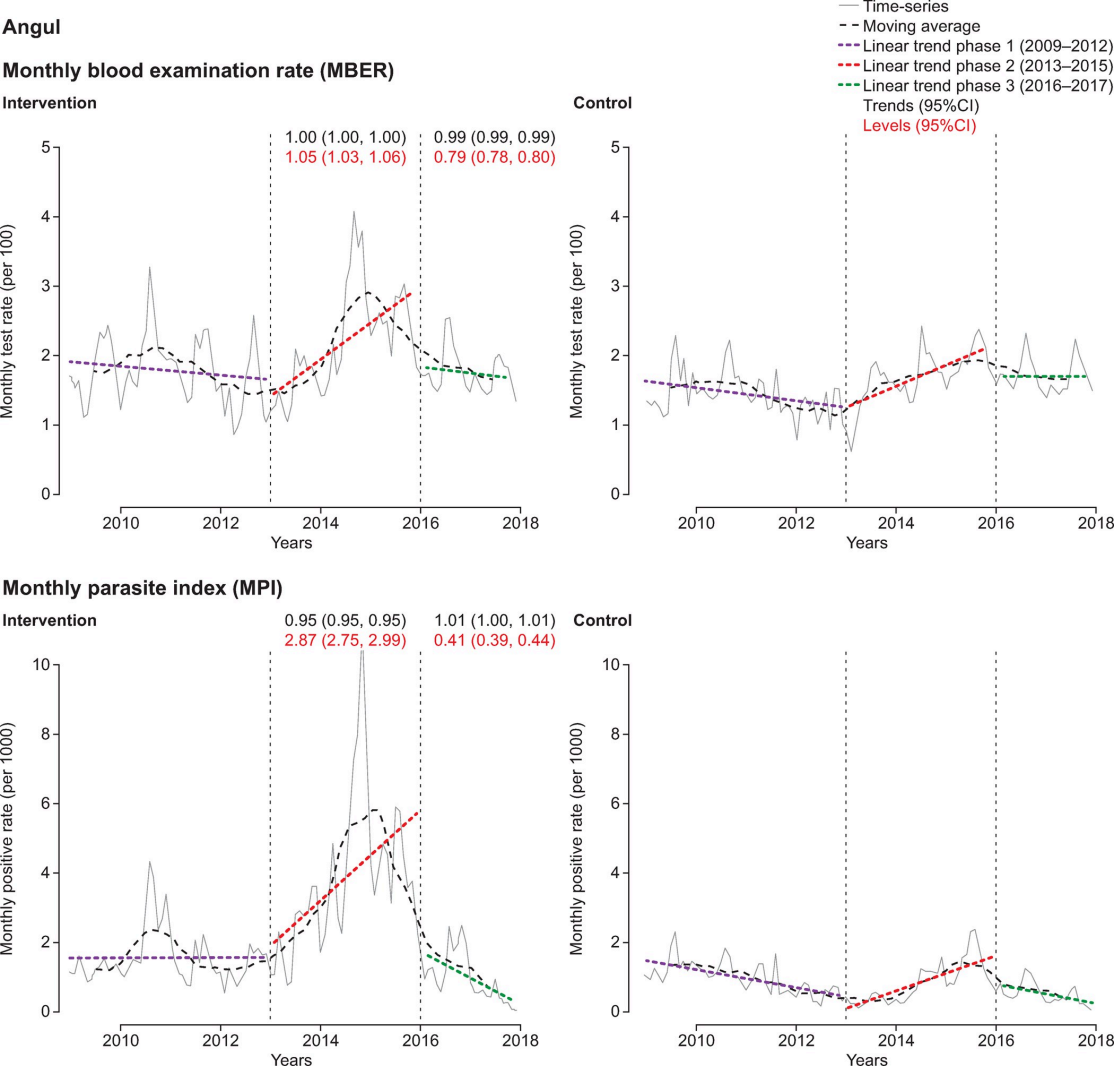
Interrupted time-series analysis of changes in malaria indices for Angul. MBER, monthly blood examination rate = total number of slides examined in a month x1000 / total population; MPI, monthly parasite index = total number of positive slides for parasite in a month x1000 / total population.

**Fig 7:**
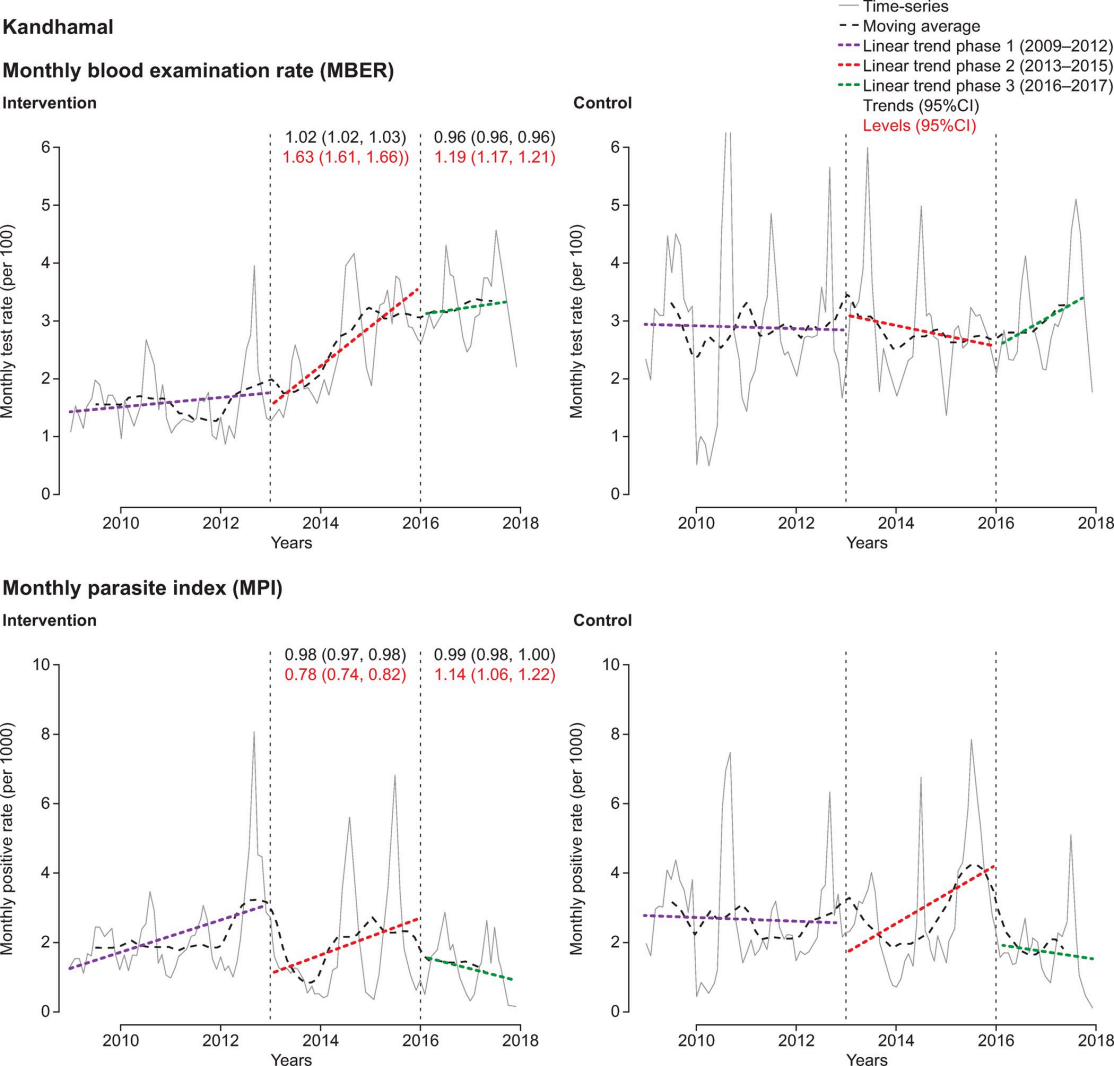
Interrupted time-series analysis of changes in malaria indices for Kandhamal. MBER, monthly blood examination rate = total number of slides examined in a month x1000 / total population; MPI, monthly parasite index = total number of positive slides for parasite in a month x1000 / total population.

The district-level ITS analysis revealed substantial differences in CCMP impact (Figs 4–7, S6 and S7 Tables). There were only small variations in the MBER and MPI trends for control-adjusted values comparing phase 1 to phase 2 and phase 2 to phase 3. However, adjusted MBER levels between phases 1 and 2 indicated a 129% (95%CI 125, 132) increase in Dhenkanal, and a 63% (95%CI 61, 66) increase in Kandhamal, but only a small change in Angul and a decline in Bolangir. For adjusted MPI levels, between phase 1 and phase 2 there was a substantial (>10-fold) increase in Dhenkanal, and nearly 3-fold increase in Angul, though MPI levels decreased in both Bolangir (−47%; 95%CI −61, −52) and in Kandhamal (−22%; 95%CI −26, −18). Between phases 2 and 3 there was a small adjusted decline in MBER levels in Bolangir (−11%; 95%CI −13, −10), a larger decline in Angul (−21%; 95%CI −22, −20), no change in Dhenkanal and an increase in Kandhamal (19%; 95%CI 17, 21). Adjusted MPI levels, declined in three districts between phase 2 and phase 3, particularly Dhenkanal (−78%; 95%CI −81, −73), but increased in Kandhamal by 14% (95%CI 6, 22).

In summary, case detection (MPI) increased overall in phase 2, primarily through *level* changes and declined remarkably in phase 3, again mainly through *level* changes, whereas the changes in the *trends* were hardly tangible. Again, the dramatic changes, first rise and then fall, were most evident in Dhenkanal and Angul.

## Discussion

Despite a trend for an overall reduction in malaria incidence in Odisha between 2003 and 2013, the malaria burden remained substantial, particularly in remote areas [13]. Previous studies on malaria intensification strategies in the state and a gap analysis of potential interventions led to the development and implementation of CCMP [13,16]. This was deployed against a background of significant investment in the ASHA network to improve malaria service provision and the availability of RDTs. These social and technical advances provided the framework to deploy EDCT more fully to remote populations in difficult-to-reach areas. CCMP interventions such as case-based tracking of malaria management was done for patients detected in intervention blocks only and was not practiced in control blocks, allowing objective assessment of the programmatic outcomes. Although conducting an operational study was challenging, evaluation of CCMP impacts across regions with differing malaria endemicity was a critical component of the programme.

Overall, both the DID and ITS analyses indicated a substantial increase in MPI during CCMP implementation, followed by a conspicuous decline in MPI post-CCMP. These changes were seen compared against an MBER that was essentially maintained with a modest increase between phases 1 and 2, and a modest decline between phases 2 and 3. This more extensive analysis confirmed the previous findings that the CCMP impact was an overall −47% decrease in malaria case incidence [16].

The current analysis revealed important differences between the four districts selected. The level changes in MPI between phase 1 and 2 were most marked in Dhenkanal (medium endemic) and Angul (high endemic) with a greater than 10-fold and nearly 3-fold increase, respectively. Similarly, between phase 2 and phase 3, despite the MBER remaining reasonably constant, the most profound decrease in MPI was in Dhenkanal (−77%), followed by Angul (−58%). As there were many inaccessible pockets without a health provider in these areas, as well as a lack of sufficient RDTs and antimalarial treatment, there was considerable scope to increase case detection through CCMP. In particular, the deployment of MSAT enabled asymptomatic cases to be identified and treated. In these difficult-to-reach hotspots for malaria transmission, asymptomatic carriage is common and represents a significant barrier to malaria elimination [12,19–22]. The final MSAT results from the CCMP are to be reported separately. We conclude that the increased case detection during CCMP along with the complete treatment of these cases led to a decline in malaria transmission and subsequent case reduction.

In contrast, Kandhamal (hyperendemic) and Bolangir (low endemic) reported control-adjusted declines in MPI from phase 1 to 2. In Bolangir, the decrease in MPI was observed alongside a decrease in MBER. In this case, it is likely that the historically low malaria transmission in Bolangir explains the lack of any detectable additional impact of CCMP. Also, in contrast to the other districts, the Bolangir control block had higher malaria risk factors than the intervention block, with a higher number of fever cases and malaria tests.

In Kandhamal, the decline in MPI from phase 1 to phase 2 occurred against a substantial increase in MBER. This can be partially explained by the saturation of the district with long-lasting insecticide impregnated bed nets (LLIN), just prior the CCMP roll out, which likely precipitated a rapid decline in malaria transmission. Also, the malaria risk factors in the Kandhamal control block were lower than in the intervention block, flattening the expected impact of CCMP. Between phase 2 and phase 3, although MBER remained stable in Kandhamal, there was a 14% increase in adjusted MPI. However, the unadjusted decrease of 33% suggests that this effect is being driven by the control block, which had a lower malaria risk factor profile versus the intervention block. The confounding effect of mass LLIN distribution before the CCMP intervention might also have contributed; the effect of LLINs was potentially diminished over the 3–4 years between their distribution in 2013 and the post-intervention phase (2016–2017), and this would be expected to increase the number of confirmed malaria cases. A study of the effectiveness and longevity of LLINs in Odisha reported that after 2 years only 57% of LLNIs were without holes, and after 4 years only 24% were without holes [23]. Bioefficacy had also weakened after four years of use, with vector mortality below 80% for 97% of the nets tested [23].

In hyperendemic areas such as Kandhamal, CCMP initiatives which focussed on increasing access to EDCT are unlikely to be sufficient to bend the case-emergence curve. Integrated malaria control, with increased attention on vector control and environmental management, as well as EDCT and extensive surveillance, is likely necessary to cause a significant impact on transmission in hyperendemic areas [22,24,25]. Note that distribution of LLINs was repeated in 2017, achieving 100% coverage of the Kandhamal population across the district, with 58% of the population covered by indoor residual spraying. In concert with these efforts, community education initiatives focused on understanding and mitigating the risks of outdoor sleeping and emphasised the need for individuals to bring their LLINs when moving between different extended family residences.

**Fig 8:**
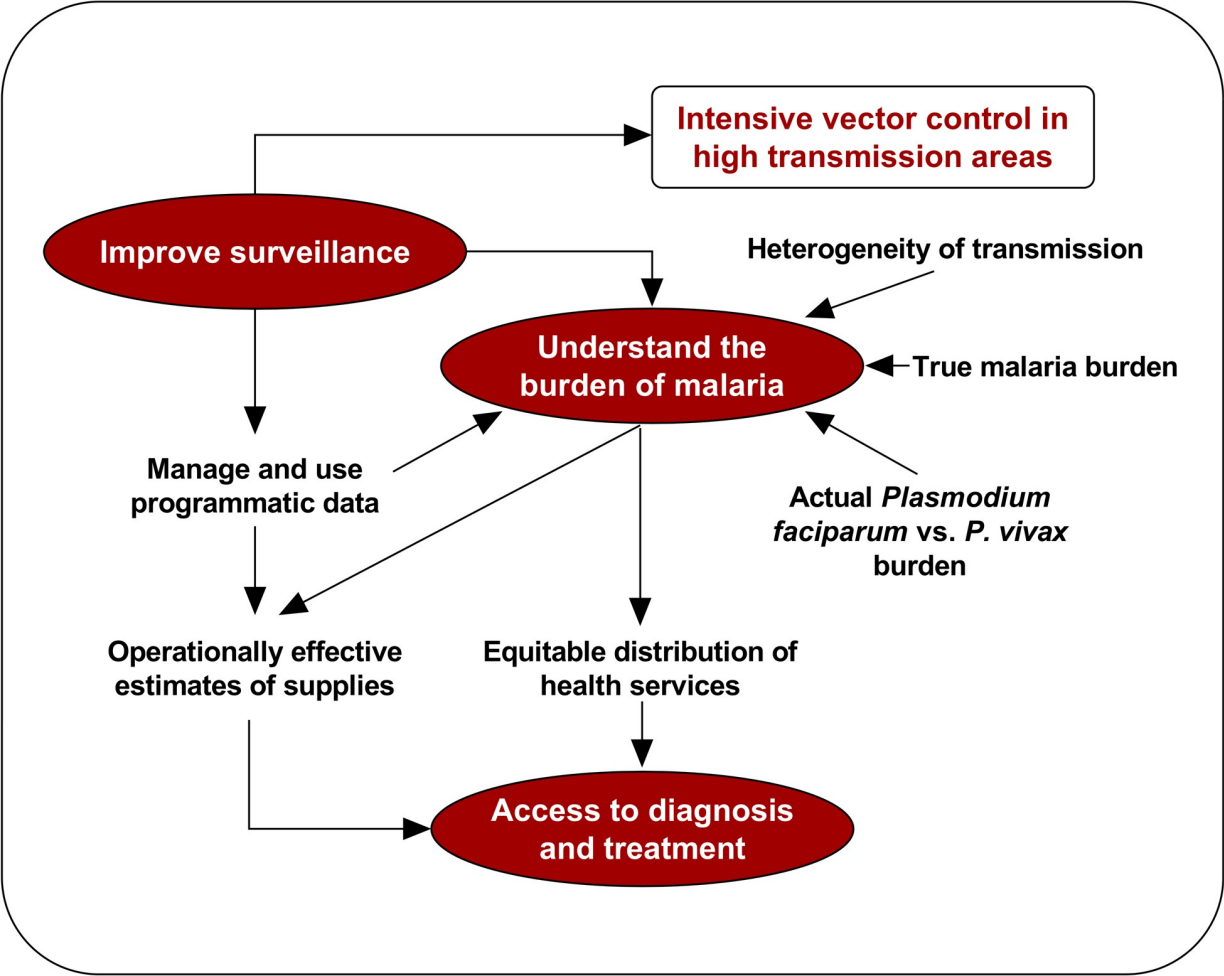
Framework for scale up of access to malaria diagnosis and treatment.

In India, the API is the key driver for targeting vector control measures. CCMP highlighted that poor surveillance leads to an underestimation of the need for malaria services, which then compounds under detection, supporting continued malaria transmission. Comprehensive case management can improve the overall understanding of the malaria burden and particularly the heterogeneity in transmission, promoting good access to EDCT. The CCMP programme in Odisha successfully demonstrated the capacity to improve data quality and reporting, as well as completeness of surveillance–thereby improving data driven decision making. The programme revealed the impact of access to diagnosis, and extended the coverage of the follow-up services required for improved treatment compliance and parasite clearance from the population at risk [15]. Ultimately, it resulted in a near halving of the malaria burden in Odisha and informed the further evolution of malaria surveillance, diagnosis, control and treatment in the region. A summary of the programmatic learning framework derived from CCMP is shown in Fig 8.

The CCMP learning framework was used to drive changes not only in the approach to malaria control and elimination in Odisha, but also to re-orientate the health system to better serve remote communities. Most significantly, in combination with the results of MSAT in hard-to reach areas, the CCMP findings contributed to the development of a state-wide project rolled out in 2017 called “Durgama Anchalare Malaria Nirakarana (DAMaN)” (English translation: “Malaria Elimination in Remote Areas”). DAMaN targeted 23 high malaria burden districts of the state, comprising ~7000 villages in hard-to-reach areas, home to around 1 million people, and included MSAT, vector control (including the promotion and distribution of LLIN), and community mobilisation to promote behavioural changes to prevent malaria and address asymptomatic carriage [12]. A key component of the CCMP and DAMaN has been capacity building of community and frontline workers like Goan Kalyan Samiti, ASHA, Auxiliary Nurse Midwifery (ANM) and Self-Help Groups (SHGs). This community-based infrastructure also targets other vector borne and water borne diseases and in 2020 was directed to include information, education and communication on the novel coronavirus. Lessons from CCMP have also been incorporated in the National Strategic Plan for Malaria Elimination [26], for example, MSAT is now recommended as an immediate response for epidemic control. Also, supply chain management has been re-oriented so that RDT and ACT supply is based on service delivery points (i.e. the number of ASHAs and community workers in an area), rather than on the number of patients tested and malaria positive cases in the previous year. Buffer stocks are also maintained at the sub-centre level to ensure RDT and ACT supply and prevent stock-outs.

There are some limitations of this study. The control blocks were matched on epidemiological indicators, but not on malaria transmission risk factors. Differences in malaria risk factors between control and intervention blocks could attenuate or accentuate the measurable effect of CCMP. However, the substantial effects that we observed are unlikely to be caused by mismatched factors only. Both the DID and ITS analyses assume that any underlying factors driving changes in the measured outcomes remain essentially the same over the analysis periods, so any deviations from the expected trend is caused by the intervention. However, it was apparent that the universal deployment of LLINs in Kandhamal just before CCMP roll-out substantially changed the malaria transmission characteristics of the district between phase 1 and phase 2, preventing the meaningful evaluation of CCMP outcomes in this district. Thus, the impact of CCMP in hyperendemic areas of Odisha cannot be determined from this analysis. However, the data do indirectly support the positive impact of LLIN distribution on reducing malaria case incidence. We did not evaluate the data separately for *Plasmodium falciparum* versus *P*. *vivax* in this study, as reliable data on the infecting species was not available for the pre-intervention period. In Odisha, *P*. *falciparum* is the predominant parasite, identified in around 87% of cases in 2014 [27]. However, *P*. *vivax* is increasing in importance in areas of low malaria transmission, and in the future it may be necessary to differentiate between the two parasites when evaluating programmatic outcomes [27]. Introduction of the bi-valent rapid diagnostic test in 2014 will allow these data to be captured going forward.

CCMP contributes to the global body of knowledge accumulating from various operational programs targeting malaria control and elimination through improved case management [28,29]. Although the situation in Odisha is unique even to India, there are parallels with other programs. For example, the CCMP strategy and structure was established by the NVBDCP, but this was essentially a decentralized program, dependent upon engagement with local communities. The need to improve the quality of community-based healthcare through enhanced training of providers and education of at-risk populations is a common theme observed elsewhere in India, as well as in Asia, Central and South America, and Africa [4–6,9–11]. A key finding of the CCMP was that improving malaria surveillance, including data capture and processing, was critical in discovering gaps in the provision of malaria services and deploying appropriate interventions. This is consistent with the WHO Technical Strategy to transform malaria surveillance into a key intervention [30]. Consequently, the introduction of timely and accurate data collection and analysis will be essential in achieving India’s malaria elimination goals [7,31]. The CCMP also employed MSAT successfully in difficult-to-reach areas. This strategy is being investigated in different settings globally as a cost-effective tool to reduce malaria disease burden and interrupt transmission, but requires careful evaluation as it is not appropriate in all circumstances [32–39].

In summary, CCMP improved EDCT coverage through the enhancement of the existing network of malaria services. This led to a dramatic increase in case detection and a subsequent decline in malaria incidence, particularly in previously difficult-to-reach communities. Malaria elimination in Odisha, and more widely India, can only be contemplated if continued transmission in underserved populations can be adequately addressed.

## Supporting information

S1 Table Malaria risk criteria of the CCMP intervention and control blocks percentage of villages. (PDF)

S2 Table Indian Census 2011 data on population composition of the study areas. (PDF)

S3 Table Study population, number of malaria tests conducted, and number of positive malaria tests by year. (PDF)

S4 Table Coverage of tracking and follow up of malaria cases with treatment cards. (PDF)

S5 Table Difference-in-difference estimates for malaria indices for the CCMP intervention. (DOCX)

S6 Table Interrupted time-series analysis of trends and levels for monthly blood examination rates. (DOCX)

S7 Table Interrupted time-series analysis of trends and levels for monthly parasite index. (DOCX)

## References

[1] World Health Organization (2021) World malaria report 2021. WHO: Geneva. Available at: https://www.who.int/teams/global-malaria-programme/reports/world-malaria-report-2021. Accessed 27 January 2022.

[2] Rae JD, Nosten S, Proux S, Myint Thu A, Cho WC, Paw K, et al. The role of monitoring and evaluation to ensure functional access to community-based early diagnosis and treatment in a malaria elimination programme in Eastern Myanmar. Malar J. 2019;18:50. doi: 10.1186/s12936-019-2677-2 30795764 PMC6387481

[3] Landier J, Parker DM, Thu AM, Carrara VI, Lwin KM, Bonnington CA, et al. The role of early detection and treatment in malaria elimination. Malar J. 2016;15:363. doi: 10.1186/s12936-016-1399-y 27421656 PMC4946177

[4] Aung PP, Thein ZW, Hein ZNM, Aung KT, Mon NO, Linn NYY, et al. Challenges in early phase of implementing the 1-3-7 surveillance and response approach in malaria elimination setting: A field study from Myanmar. Infect Dis Poverty. 2020;9:18. doi: 10.1186/s40249-020-0632-7 32036792 PMC7008564

[5] Rajvanshi H, Mishra K, Bharti PK, Sandhibigraha D, Nisar S, Jayswar H, et al. Learnings from two independent malaria elimination demonstration projects in India. Trans R Soc Trop Med Hyg. 2021;115:1229–1233. doi: 10.1093/trstmh/trab148 34563095

[6] Napier HG, Baird M, Wong E, Walwyn-Jones E, Garcia ME, Cartagena L, et al. Evaluating vertical malaria community health worker programs as malaria declines: Learning from program evaluations in Honduras and Lao PDR. Glob Health Sci Pract. 2021;9:S98–S110. doi: 10.9745/GHSP-D-20-00379 33727323 PMC7971372

[7] Baliga BS, Baliga S, Jain A, Kulal N, Kumar M, Koduvattat N, et al. Digitized smart surveillance and micromanagement using information technology for malaria elimination in Mangaluru, India: an analysis of five-year post-digitization data. Malar J. 2021;20:139. doi: 10.1186/s12936-021-03656-8 33685454 PMC7938374

[8] Rajvanshi H, Bharti PK, Nisar S, Jayswar H, Mishra AK, Sharma RK, et al. A model for malaria elimination based on learnings from the Malaria Elimination Demonstration Project, Mandla district, Madhya Pradesh. Malar J. 2021;20:98. doi: 10.1186/s12936-021-03607-3 33593368 PMC7888092

[9] Prat JGI, Morais P, Claret M, Badia P, Fialho RR, Albajar-Vinas P, et al. Community-based approaches for malaria case management in remote communities in the Brazilian Amazon. Rev Soc Bras Med Trop. 2020;53:e20200048. doi: 10.1590/0037-8682-0048-2020 32997048 PMC7514773

[10] Galatas B, Saute F, Marti-Soler H, Guinovart C, Nhamussua L, Simone W, et al. A multiphase program for malaria elimination in southern Mozambique (the Magude project): A before-after study. PLoS Med. 2020;17:e1003227. doi: 10.1371/journal.pmed.1003227 32797101 PMC7428052

[11] Mlacha YP, Wang D, Chaki PP, Gavana T, Zhou Z, Michael MG, et al. Effectiveness of the innovative 1,7-malaria reactive community-based testing and response (1, 7-mRCTR) approach on malaria burden reduction in Southeastern Tanzania. Malar J. 2020;19:292. doi: 10.1186/s12936-020-03363-w 32799857 PMC7429894

[12] Bal M, Das A, Ghosal J, Pradhan MM, Khuntia HK, Pati S, et al. Assessment of effectiveness of DAMaN: A malaria intervention program initiated by Government of Odisha, India. PLoS One. 2020;15:e0238323. doi: 10.1371/journal.pone.0238323 32898853 PMC7478908

[13] Pradhan A, Anasuya A, Pradhan MM, Ak K, Kar P, Sahoo KC, et al. Trends in malaria in Odisha, India—an analysis of the 2003–2013 time-series data from the National Vector Borne Disease Control Program. PLoS One. 2016;11:e0149126. doi: 10.1371/journal.pone.0149126 26866696 PMC4750863

[14] Pradhan MM, Meherda PK. Malaria elimination drive in Odisha: Hope for halting the transmission. J Vector Borne Dis. 2019;56:53–55. doi: 10.4103/0972-9062.257775 31070166

[15] Pradhan S, Pradhan MM, Dutta A, Shah NK, Joshi PL, Pradhan K, et al. Improved access to early diagnosis and complete treatment of malaria in Odisha, India. PLoS One. 2019;14:e0208943. doi: 10.1371/journal.pone.0208943 30601833 PMC6314604

[16] Pradhan MM, Anvikar AR, Daumerie PG, Pradhan S, Dutta A, Shah NK, et al. Comprehensive case management of malaria: Operational research informing policy. J Vector Borne Dis. 2019;56:56–59. doi: 10.4103/0972-9062.257776 31070167

[17] Anvikar AR, Arora U, Sonal GS, Mishra N, Shahi B, Savargaonkar D, et al. Antimalarial drug policy in India: past, present & future. Indian J Med Res. 2014;139:205–215. 24718394 PMC4001331

[18] Ramsay CR, Matowe L, Grilli R, Grimshaw JM, Thomas RE. Interrupted time series designs in health technology assessment: lessons from two systematic reviews of behavior change strategies. Int J Technol Assess Health Care. 2003;19:613–623. doi: 10.1017/s0266462303000576 15095767

[19] Kumari P, Sinha S, Gahtori R, Yadav CP, Pradhan MM, Rahi M, et al. Prevalence of asymptomatic malaria parasitemia in Odisha, India: A challenge to malaria elimination. Am J Trop Med Hyg. 2020;103:1510–1516. doi: 10.4269/ajtmh.20-0018 32783792 PMC7543830

[20] Panda B, Mohapatra MK, Paital S, Kumbhakar S, Dutta A, Kadam S, et al. Prevalence of afebrile malaria and development of risk-scores for gradation of villages: A study from a hot-spot in Odisha. PLoS One. 2019;14:e0221223. doi: 10.1371/journal.pone.0221223 31490940 PMC6730888

[21] van Eijk AM, Sutton PL, Ramanathapuram L, Sullivan SA, Kanagaraj D, Priya GSL, et al. The burden of submicroscopic and asymptomatic malaria in India revealed from epidemiology studies at three varied transmission sites in India. Sci Rep. 2019;9:17095. doi: 10.1038/s41598-019-53386-w 31745160 PMC6863831

[22] MalERA Refresh Consultative Panel on Characterising the Reservoir and Measuring Transmission. malERA: An updated research agenda for characterising the reservoir and measuring transmission in malaria elimination and eradication. PLoS Med. 2017;14:e1002452. doi: 10.1371/journal.pmed.1002452 29190279 PMC5708619

[23] Anuse SS, Sahu SS, Subramanian S, Gunasekaran K. Usage pattern, physical integrity & insecticidal efficacy of long-lasting insecticidal nets in Odisha State, India. Indian J Med Res. 2015;142 Suppl:S71–78. 26905246 10.4103/0971-5916.176628 PMC4795351

[24] MalERA Refresh Consultative Panel on Combination Interventions and Modelling. malERA: An updated research agenda for combination interventions and modelling in malaria elimination and eradication. PLoS Med. 2017;14:e1002453. doi: 10.1371/journal.pmed.1002453 29190295 PMC5708628

[25] Rabinovich RN, Drakeley C, Djimde AA, Hall BF, Hay SI, Hemingway J, et al. malERA: An updated research agenda for malaria elimination and eradication. PLoS Med. 2017;14:e1002456. doi: 10.1371/journal.pmed.1002456 29190300 PMC5708604

[26] National Vector Borne Disease Control Programme National strategic plan: Malaria elimination in India 2017–2022. Government of India: Delhi. Available at: http://www.indiaenvironmentportal.org.in/files/file/nsp_2017-2022-updated.pdf. Accessed 14 June 2021.

[27] Anvikar AR, Shah N, Dhariwal AC, Sonal GS, Pradhan MM, Ghosh SK, et al. Epidemiology of *Plasmodium vivax* malaria in India. Am J Trop Med Hyg. 2016;95:108–120. doi: 10.4269/ajtmh.16-0163 27708188 PMC5201217

[28] Smith Gueye C, Newby G, Tulloch J, Slutsker L, Tanner M, Gosling RD. The central role of national programme management for the achievement of malaria elimination: a cross case-study analysis of nine malaria programmes. Malar J. 2016;15:488. doi: 10.1186/s12936-016-1518-9 27659770 PMC5034437

[29] Shretta R, Liu J, Cotter C, Cohen J, Dolenz C, Makomva K, et al. (2017) Malaria elimination and eradication. In: Holmes KK BS, Bloom BR, et al., editor. Major Infectious Diseases (third edition). Washington (DC): The World Bank. 30212099

[30] World Health Organization (2015) Global technical strategy for malaria 2016–2030. WHO: Geneva. Available at: https://www.who.int/docs/default-source/documents/global-technical-strategy-for-malaria-2016-2030.pdf. Accessed 26 Jan 2022.

[31] Rahi M, Sharma A. For malaria elimination India needs a platform for data integration. BMJ Glob Health. 2020;5. 10.1136/bmjgh-2020-004198 PMC7780526 33380414

[32] Rajvanshi H, Bharti PK, Nisar S, Jain Y, Jayswar H, Mishra AK, et al. Study design and operational framework for a community-based Malaria Elimination Demonstration Project (MEDP) in 1233 villages of district Mandla, Madhya Pradesh. Malar J. 2020;19:410. doi: 10.1186/s12936-020-03458-4 33198754 PMC7667481

[33] Bharti PK, Rajvanshi H, Nisar S, Jayswar H, Saha KB, Shukla MM, et al. Demonstration of indigenous malaria elimination through Track-Test-Treat-Track (T4) strategy in a Malaria Elimination Demonstration Project in Mandla, Madhya Pradesh. Malar J. 2020;19:339. doi: 10.1186/s12936-020-03402-6 32943065 PMC7499908

[34] Lek D, Callery JJ, Nguon C, Debackere M, Sovannaroth S, Tripura R, et al. Tools to accelerate falciparum malaria elimination in Cambodia: a meeting report. Malar J. 2020;19:151. doi: 10.1186/s12936-020-03197-6 32293452 PMC7161105

[35] Mendis K. Mass drug administration should be implemented as a tool to accelerate elimination: against. Malar J. 2019;18:279. doi: 10.1186/s12936-019-2907-7 31438943 PMC6704542

[36] Bousema T, Stresman G, Baidjoe AY, Bradley J, Knight P, Stone W, et al. The impact of hotspot-targeted interventions on malaria transmission in Rachuonyo South District in the Western Kenyan highlands: A cluster-randomized controlled trial. PLoS Med. 2016;13:e1001993. doi: 10.1371/journal.pmed.1001993 27071072 PMC4829260

[37] Linn AM, Ndiaye Y, Hennessee I, Gaye S, Linn P, Nordstrom K, et al. Reduction in symptomatic malaria prevalence through proactive community treatment in rural Senegal. Trop Med Int Health. 2015;20:1438–1446. doi: 10.1111/tmi.12564 26171642

[38] Silumbe K, Yukich JO, Hamainza B, Bennett A, Earle D, Kamuliwo M, et al. Costs and cost-effectiveness of a large-scale mass testing and treatment intervention for malaria in Southern Province, Zambia. Malar J. 2015;14:211. doi: 10.1186/s12936-015-0722-3 25985992 PMC4490652

[39] Crowell V, Briet OJ, Hardy D, Chitnis N, Maire N, Di Pasquale A, et al. Modelling the cost-effectiveness of mass screening and treatment for reducing *Plasmodium falciparum* malaria burden. Malar J. 2013;12:4. doi: 10.1186/1475-2875-12-4 23286228 PMC3544609

