# Supplementary material for: Impact of the malaria comprehensive case management programme in Odisha, India
Source: PLoS One. 2022 Mar 24;17(3):e0265352. doi: 10.1371/journal.pone.0265352 (PMC8947122; doi:10.1371/journal.pone.0265352)
Supplement: S1 Table — (PDF) [file pone.0265352.s001.pdf]

S1 Table Malaria risk criteria of the CCMP intervention and control blocks percentage of villages.

Factor	Criteria	Bolangir		Dhenkanal		Angul		Kandhamal		Overall	
		CCMP (N=145)	Control (N=156)	CCMP (N=207)	Control (N=94)	CCMP (N=302)	Control (N=188)	CCMP (N=304)	Control (N=262)	CCMP (N=958)	Control (N=700)
Road access, %	Good road	78	80	92	94	59	68	31	63	61	72
	Inaccessible in rainy season	21	16	7	7	33	28	8	22	17	20
	Only footpaths	1	5	1	0	9	5	61	15	22	8
Forest coverage, %	No forest	98	52	84	95	17	39	0	43	40	51
	Open forest < 40%	3	33	7	5	36	42	32	38	23	33
	Dense forest > 70%	0	16	7	0	47	19	68	19	37	16
Streams, %	None	100	66	91	93	46	63	1	83	52	76
	Intermittent	0	24	0	2	22	18	94	12	35	15
	Perennial	0	10	9	6	32	19	5	5	14	10
Irrigation, %	No	100	99	48	82	82	90	0	0	53	56
	Yes	0	1	52	18	18	10	100	100	48	43
Geophysical position, %	Plain	99	80	89	93	58	81	5	30	56	63
	Surrounded by forest	0	16	6	4	28	11	2	24	11	16
	Foothills	1	4	5	4	14	8	93	46	34	21
Cultivation, %	No	0	0	0	0	38	4	0	1	12	1
	Yes	100	100	100	100	62	96	100	99	88	99
Distance from diagnosis, km		0.8	0.5	0.8	0.6	1.8	1.2	2.7	1.4	1.7	1.0

CCMP, Comprehensive Case Management Project.
