# Supplementary material for: Impact of the malaria comprehensive case management programme in Odisha, India
Source: PLoS One. 2022 Mar 24;17(3):e0265352. doi: 10.1371/journal.pone.0265352 (PMC8947122; doi:10.1371/journal.pone.0265352)
Supplement: S2 Table — (PDF) [file pone.0265352.s002.pdf]

S2 Table Difference-in-difference estimates for malaria indices for the CCMP intervention.

Indices	Block	Analysis	Comparison	Bolangir	Dhenkanal	Angul	Kandhamal	Pooled
MBER	CCMP intervention	Unadjusted	Phase 1 vs. 2	1.27 (1.25, 1.28)	1.97 (1.95, 2.00)	1.23 (1.22, 1.24)	1.60 (1.58, 1.62)	1.46 (1.45, 1.47)
			Phase 2 vs. 3	1.00 (0.98, 1.01)	1.06 (1.05, 1.07)	0.80 (0.79, 0.81)	1.27 (1.26, 1.29)	1.00 (0.99, 1.00)
		Adjusted for control	Phase 1 vs. 2	0.73 (0.72, 0.74)	2.28 (2.25, 2.32)	1.04 (1.03, 1.06)	1.63 (1.61, 1.66)	1.25 (1.24, 1.26)
			Phase 2 vs. 3	0.89 (0.87, 0.90)	1.01 (1.00, 1.03)	0.80 (0.78, 0.81)	1.19 (1.17, 1.21)	0.96 (0.96, 0.97)
	Control		Phase 1 vs. 2	1.74 (1.72, 1.76)	0.86 (0.85, 0.88)	1.18 (1.17, 1.19)	0.98 (0.97, 0.99)	1.16 (1.16, 1.17)
			Phase 2 vs. 3	1.12 (1.11, 1.14)	1.04 (1.03, 1.06)	1.00 (1.00, 1.01)	1.07 (1.05, 1.08)	1.03 (1.03, 1.04)
MPI	CCMP intervention	Unadjusted	Phase 1 vs. 2	0.59 (0.53, 0.65)	2.42 (2.31, 2.54)	2.45 (2.39, 2.52)	0.86 (0.82, 0.89)	1.83 (1.79, 1.86)
			Phase 2 vs. 3	0.61 (0.53, 0.71)	0.17 (0.16, 0.19)	0.27 (0.26, 0.28)	0.67 (0.64, 0.71)	0.32 (0.31, 0.33)
		Adjusted for control	Phase 1 vs. 2	0.43 (0.38, 0.48)	10.71 (9.56, 12.03)	2.86 (2.73, 2.98)	0.78 (0.74, 0.82)	1.96 (1.90, 2.02)
			Phase 2 vs. 3	0.87 (0.74, 1.03)	0.23 (0.19, 0.27)	0.42 (0.39, 0.45)	1.14 (1.06, 1.22)	0.53 (0.51, 0.55)
	Control		Phase 1 vs. 2	1.37 (1.29, 1.45)	0.23 (0.20, 0.25)	0.86 (0.83, 0.89)	1.10 (1.06, 1.14)	0.93 (0.91, 0.95)
			Phase 2 vs. 3	0.70 (0.65, 0.75)	0.77 (0.67, 0.89)	0.63 (0.60, 0.66)	0.59 (0.57, 0.62)	0.60 (0.58, 0.62)

Data are estimates (95%CI). Phase 1 = pre-CCMP; Phase 2 = CCMP intervention; Phase 3 = post-CCMP.

CCMP, Comprehensive Case Management Project; MBER, monthly blood examination rate = total number of slides examined in a month x1000 / total population; MPI, monthly parasite index = total number of positive slides for parasite in a month x1000 / total population.
