# Supplementary material for: Impact of the malaria comprehensive case management programme in Odisha, India
Source: PLoS One. 2022 Mar 24;17(3):e0265352. doi: 10.1371/journal.pone.0265352 (PMC8947122; doi:10.1371/journal.pone.0265352)
Supplement: S3 Table — (PDF) [file pone.0265352.s003.pdf]

**S3 Table Interrupted time-series analysis of trends and levels for monthly blood examination rates.**

| Indices | Block | Analysis | Comparison | Bolangir | Dhenkanal | Angul | Kandhamal | Pooled |
|---|---|---|---|---|---|---|---|---|
| Trends | CCMP intervention | Unadjusted | Phase 1 vs. 2 | 1.00 (1.00, 1.00) | 1.03 (1.03, 1.03) | 1.02 (1.02, 1.02) | 1.02 (1.02, 1.02) | 1.02 (1.02, 1.02) |
| | | | Phase 2 vs. 3 | 0.97 (0.97, 0.97) | 0.97 (0.97, 0.97) | 0.98 (0.97, 0.98) | 0.98 (0.98, 0.98) | 0.97 (0.97, 0.98) |
| | | Adjusted for control | Phase 1 vs. 2 | 0.99 (0.99, 0.99) | 1.00 (1.00, 1.00) | 1.00 (1.00, 1.00) | 1.02 (1.02, 1.03) | 1.01 (1.00, 1.01) |
| | | | Phase 2 vs. 3 | 1.00 (1.00, 1.01) | 1.00 (1.00, 1.00) | 0.99 (0.99, 0.99) | 0.96 (0.96, 0.96) | 0.99 (0.99, 0.99) |
| | Control | | Phase 1 vs. 2 | 1.01 (1.01, 1.01) | 1.03 (1.03, 1.03) | 1.02 (1.02, 1.02) | 1.00 (0.99, 1.00) | 1.01 (1.01, 1.02) |
| | | | Phase 2 vs. 3 | 0.97 (0.97, 0.97) | 0.97 (0.97, 0.97) | 0.99 (0.98, 0.99) | 1.02 (1.02, 1.02) | 0.98 (0.98, 0.98) |
| Levels | CCMP intervention | Unadjusted | Phase 1 vs. 2 | 0.89 (0.87, 0.91) | 1.47 (1.44, 1.50) | 0.90 (0.89, 0.92) | 0.95 (0.92, 0.97) | 1.01 (1.00, 1.02) |
| | | | Phase 2 vs. 3 | 1.04 (1.02, 1.07) | 0.79 (0.77, 0.80) | 0.60 (0.59, 0.61) | 0.85 (0.83, 0.87) | 0.77 (0.77, 0.78) |
| | | Adjusted for control | Phase 1 vs. 2 | 0.73 (0.72, 0.74) | 2.29 (2.25, 2.32) | 1.05 (1.03, 1.06) | 1.63 (1.61, 1.66) | 1.25 (1.24, 1.26) |
| | | | Phase 2 vs. 3 | 0.89 (0.87, 0.90) | 1.01 (0.99, 1.02) | 0.79 (0.78, 0.80) | 1.19 (1.17, 1.21) | 0.96 (0.96, 0.97) |
| | Control | | Phase 1 vs. 2 | 0.99 (0.96, 1.01) | 0.78 (0.76, 0.80) | 1.02 (1.00, 1.04) | 1.09 (1.07, 1.11) | 0.98 (0.97, 0.99) |
| | | | Phase 2 vs. 3 | 0.94 (0.92, 0.96) | 0.90 (0.88, 0.92) | 0.79 (0.77, 0.80) | 0.99 (0.97, 1.02) | 0.98 (0.98, 0.98) |

Data are difference-in-difference estimates (95%CI). Monthly blood examination rate = total number of slides examined in a month x1000 / total population. Phase 1 = pre-CCMP; Phase 2 = CCMP intervention; Phase 3 = post-CCMP.

CCMP, Comprehensive Case Management Project.
