# Supplementary material for: Impact of the malaria comprehensive case management programme in Odisha, India
Source: PLoS One. 2022 Mar 24;17(3):e0265352. doi: 10.1371/journal.pone.0265352 (PMC8947122; doi:10.1371/journal.pone.0265352)
Supplement: S4 Table — (PDF) [file pone.0265352.s004.pdf]

S4 Table Interrupted time-series analysis of trends and levels for monthly parasite index.

Indices	Block	Analysis	Comparison	Bolangir	Dhenkanal	Angul	Kandhamal	Pooled
Trends	CCMP intervention	Unadjusted	Phase 1 vs. 2	1.02 (1.01, 1.03)	1.10 (1.10, 1.11)	1.03 (1.03, 1.03)	1.01 (1.00, 1.01)	1.03 (1.03, 1.04)
			Phase 2 vs. 3	0.97 (0.95, 0.99)	0.94 (0.93, 0.96)	0.91 (0.91, 0.92)	0.95 (0.95, 0.96)	0.93 (0.92, 0.93)
		Adjusted for control	Phase 1 vs. 2	0.98 (0.97, 0.99)	1.02 (1.00, 1.03)	0.95 (0.95, 0.95)	0.98 (0.97, 0.98)	0.98 (0.98, 0.98)
			Phase 2 vs. 3	1.04 (1.02, 1.06)	1.05 (1.02, 1.07)	1.01 (1.00, 1.01)	0.99 (0.98, 1.00)	0.99 (0.99, 1.00)
	Control		Phase 1 vs. 2	1.05 (1.04, 1.05)	1.09 (1.08, 1.10)	1.08 (1.08, 1.09)	1.03 (1.02, 1.03)	1.06 (1.05, 1.06)
			Phase 2 vs. 3	0.93 (0.92, 0.94)	0.90 (0.89, 0.92)	0.91 (0.90, 0.91)	0.97 (0.96, 0.97)	0.93 (0.93, 0.94)
Levels	CCMP intervention	Unadjusted	Phase 1 vs. 2	0.89 (0.73, 1.08)	2.91 (2.56, 3.32)	1.40 (1.33, 1.48)	0.37 (0.34, 0.40)	1.01 (0.97, 1.05)
			Phase 2 vs. 3	0.80 (0.61, 1.04)	0.08 (0.07, 0.10)	0.31 (0.30, 0.33)	0.60 (0.54, 0.66)	0.30 (0.29, 0.32)
		Adjusted for control	Phase 1 vs. 2	0.43 (0.39, 0.48)	10.76 (9.61, 12.08)	2.87 (2.75, 2.99)	0.78 (0.74, 0.82)	1.96 (1.91, 2.02)
			Phase 2 vs. 3	0.87 (0.74, 1.03)	0.22 (0.19, 0.27)	0.41 (0.39, 0.44)	1.14 (1.06, 1.22)	0.53 (0.50, 0.55)
	Control		Phase 1 vs. 2	1.28 (1.12, 1.46)	0.53 (0.41, 0.67)	0.46 (0.42, 0.51)	0.71 (0.66, 0.76)	0.69 (0.65, 0.72)
			Phase 2 vs. 3	0.71 (0.62, 0.80)	0.95 (0.74, 1.23)	0.43 (0.39, 0.47)	0.45 (0.41, 0.49)	0.48 (0.45, 0.50)

Data are difference-in-difference estimates (95%CI). Monthly parasite index = total number of positive slides for parasite in a month x1000 / total population. Phase 1 = pre-CCMP; Phase 2 = CCMP intervention; Phase 3 = post-CCMP.

CCMP, Comprehensive Case Management Project.
