# Supplementary material for: Impact of the malaria comprehensive case management programme in Odisha, India
Source: PLoS One. 2022 Mar 24;17(3):e0265352. doi: 10.1371/journal.pone.0265352 (PMC8947122; doi:10.1371/journal.pone.0265352)
Supplement: S5 Table — (DOCX) [file pone.0265352.s005.docx]

**S5 Table Difference-in-difference estimates for malaria indices for the CCMP intervention.**

| **Indices** | **Block** | **Analysis** | **Comparison** | **Bolangir** | **Dhenkanal** | **Angul** | **Kandhamal** | **Pooled** |
| --- | --- | --- | --- | --- | --- | --- | --- | --- |
| MBER | CCMP intervention | Unadjusted | Phase 1 vs. 2 | 1.27 (1.25, 1.28) | 1.97 (1.95, 2.00) | 1.23 (1.22, 1.24) | 1.60 (1.58, 1.62) | 1.46 (1.45, 1.47) |
|  |  |  | Phase 2 vs. 3 | 1.00 (0.98, 1.01) | 1.06 (1.05, 1.07) | 0.80 (0.79, 0.81) | 1.27 (1.26, 1.29) | 1.00 (0.99, 1.00) |
|  |  | Adjusted for control | Phase 1 vs. 2 | 0.73 (0.72, 0.74) | 2.28 (2.25, 2.32) | 1.04 (1.03, 1.06) | 1.63 (1.61, 1.66) | 1.25 (1.24, 1.26) |
|  |  |  | Phase 2 vs. 3 | 0.89 (0.87, 0.90) | 1.01 (1.00, 1.03) | 0.80 (0.78, 0.81) | 1.19 (1.17, 1.21) | 0.96 (0.96, 0.97) |
|  | Control |  | Phase 1 vs. 2 | 1.74 (1.72, 1.76) | 0.86 (0.85, 0.88) | 1.18 (1.17, 1.19) | 0.98 (0.97, 0.99) | 1.16 (1.16, 1.17) |
|  |  |  | Phase 2 vs. 3 | 1.12 (1.11, 1.14) | 1.04 (1.03, 1.06) | 1.00 (1.00, 1.01) | 1.07 (1.05, 1.08) | 1.03 (1.03, 1.04) |
| MPI | CCMP intervention | Unadjusted | Phase 1 vs. 2 | 0.59 (0.53, 0.65) | 2.42 (2.31, 2.54) | 2.45 (2.39, 2.52) | 0.86 (0.82, 0.89) | 1.83 (1.79, 1.86) |
|  |  |  | Phase 2 vs. 3 | 0.61 (0.53, 0.71) | 0.17 (0.16, 0.19) | 0.27 (0.26, 0.28) | 0.67 (0.64, 0.71) | 0.32 (0.31, 0.33) |
|  |  | Adjusted for control | Phase 1 vs. 2 | 0.43 (0.38, 0.48) | 10.71 (9.56, 12.03) | 2.86 (2.73, 2.98) | 0.78 (0.74, 0.82) | 1.96 (1.90, 2.02) |
|  |  |  | Phase 2 vs. 3 | 0.87 (0.74, 1.03) | 0.23 (0.19, 0.27) | 0.42 (0.39, 0.45) | 1.14 (1.06, 1.22) | 0.53 (0.51, 0.55) |
|  | Control |  | Phase 1 vs. 2 | 1.37 (1.29, 1.45) | 0.23 (0.20, 0.25) | 0.86 (0.83, 0.89) | 1.10 (1.06, 1.14) | 0.93 (0.91, 0.95) |
|  |  |  | Phase 2 vs. 3 | 0.70 (0.65, 0.75) | 0.77 (0.67, 0.89) | 0.63 (0.60, 0.66) | 0.59 (0.57, 0.62) | 0.60 (0.58, 0.62) |

Data are estimates (95%CI). Phase 1 = pre-CCMP; Phase 2 = CCMP intervention; Phase 3 = post-CCMP.

CCMP, Comprehensive Case Management Project; MBER, monthly blood examination rate = total number of slides examined in a month x1000 / total population; MPI, monthly parasite index = total number of positive slides for parasite in a month x1000 / total population.
