# Supplementary material for: Impact of the malaria comprehensive case management programme in Odisha, India
Source: PLoS One. 2022 Mar 24;17(3):e0265352. doi: 10.1371/journal.pone.0265352 (PMC8947122; doi:10.1371/journal.pone.0265352)
Supplement: S7 Table — (DOCX) [file pone.0265352.s007.docx]

**S7 Table Interrupted time-series analysis of trends and levels for monthly parasite index.**

| **Indices** | **Block** | **Analysis** | **Comparison** | **Bolangir** | **Dhenkanal** | **Angul** | **Kandhamal** | **Pooled** |
| --- | --- | --- | --- | --- | --- | --- | --- | --- |
| Trends | CCMP intervention | Unadjusted | Phase 1 vs. 2 | 1.02 (1.01, 1.03) | 1.10 (1.10, 1.11) | 1.03 (1.03, 1.03) | 1.01 (1.00, 1.01) | 1.03 (1.03, 1.04) |
|  |  |  | Phase 2 vs. 3 | 0.97 (0.95, 0.99) | 0.94 (0.93, 0.96) | 0.91 (0.91, 0.92) | 0.95 (0.95, 0.96) | 0.93 (0.92, 0.93) |
|  |  | Adjusted for control | Phase 1 vs. 2 | 0.98 (0.97, 0.99) | 1.02 (1.00, 1.03) | 0.95 (0.95, 0.95) | 0.98 (0.97, 0.98) | 0.98 (0.98, 0.98) |
|  |  |  | Phase 2 vs. 3 | 1.04 (1.02, 1.06) | 1.05 (1.02, 1.07) | 1.01 (1.00, 1.01) | 0.99 (0.98, 1.00) | 0.99 (0.99, 1.00) |
|  | Control |  | Phase 1 vs. 2 | 1.05 (1.04, 1.05) | 1.09 (1.08, 1.10) | 1.08 (1.08, 1.09) | 1.03 (1.02, 1.03) | 1.06 (1.05, 1.06) |
|  |  |  | Phase 2 vs. 3 | 0.93 (0.92, 0.94) | 0.90 (0.89, 0.92) | 0.91 (0.90, 0.91) | 0.97 (0.96, 0.97) | 0.93 (0.93, 0.94) |
| Levels | CCMP intervention | Unadjusted | Phase 1 vs. 2 | 0.89 (0.73, 1.08) | 2.91 (2.56, 3.32) | 1.40 (1.33, 1.48) | 0.37 (0.34, 0.40) | 1.01 (0.97, 1.05) |
|  |  |  | Phase 2 vs. 3 | 0.80 (0.61, 1.04) | 0.08 (0.07, 0.10) | 0.31 (0.30, 0.33) | 0.60 (0.54, 0.66) | 0.30 (0.29, 0.32) |
|  |  | Adjusted for control | Phase 1 vs. 2 | 0.43 (0.39, 0.48) | 10.76 (9.61, 12.08) | 2.87 (2.75, 2.99) | 0.78 (0.74, 0.82) | 1.96 (1.91, 2.02) |
|  |  |  | Phase 2 vs. 3 | 0.87 (0.74, 1.03) | 0.22 (0.19, 0.27) | 0.41 (0.39, 0.44) | 1.14 (1.06, 1.22) | 0.53 (0.50, 0.55) |
|  | Control |  | Phase 1 vs. 2 | 1.28 (1.12, 1.46) | 0.53 (0.41, 0.67) | 0.46 (0.42, 0.51) | 0.71 (0.66, 0.76) | 0.69 (0.65, 0.72) |
|  |  |  | Phase 2 vs. 3 | 0.71 (0.62, 0.80) | 0.95 (0.74, 1.23) | 0.43 (0.39, 0.47) | 0.45 (0.41, 0.49) | 0.48 (0.45, 0.50) |

Data are difference-in-difference estimates (95%CI). Monthly parasite index = total number of positive slides for parasite in a month x1000 / total population. Phase 1 = pre-CCMP; Phase 2 = CCMP intervention; Phase 3 = post-CCMP.

CCMP, Comprehensive Case Management Project.
